# Relief of neuropathic pain by cell-specific manipulation of nucleus accumbens dopamine D1- and D2-receptor-expressing neurons

**DOI:** 10.1186/s13041-021-00896-2

**Published:** 2022-01-06

**Authors:** Daisuke Sato, Michiko Narita, Yusuke Hamada, Tomohisa Mori, Kenichi Tanaka, Hideki Tamura, Akihiro Yamanaka, Ryosuke Matsui, Dai Watanabe, Yukari Suda, Emiko Senba, Moe Watanabe, Edita Navratilova, Frank Porreca, Naoko Kuzumaki, Minoru Narita

**Affiliations:** 1 Department of Pharmacology, Hoshi University School of Pharmacy and Pharmaceutical Sciences, 2-4-41 Ebara, Shinagawa-ku, 142-8501 Tokyo, Japan; 2 Division of Cancer Pathophysiology, National Cancer Center Research Institute (NCCRI), 5-1-1 Tsukiji, Chuo-ku, 104-0045 Tokyo, Japan; 3 Department of Molecular and Cellular Medicine, Institute of Medical science, Tokyo Medical University, 6-7-1 Nishishinjuku, Shinjuku-ku, 160-0023 Tokyo, Japan; 4 Institute for Advanced Life Sciences, Hoshi University School of Pharmacy and Pharmaceutical Sciences, 2-4-41 Ebara, Shinagawa-ku, 142-0063 Tokyo, Japan; 5 Laboratory of Biofunctional Science, Hoshi University School of Pharmacy and Pharmaceutical Sciences, 2-4-41 Ebara, Shinagawa-ku, 142-0063 Tokyo, Japan; 6 Department of Neuroscience II, Research Institute of Environmental Medicine, Nagoya University, Furo-cho, Chikusa-ku, 464-8601 Nagoya, Japan; 7 Department of Biological Sciences, Graduate school of Medicine, Kyoto University, Yoshida, Sakyo-ku, 606-8501 Kyoto, Japan; 8 Department of Physical Therapy, Osaka Yukioka College of Health Science, 1-1-41 Sojiji, Ibaraki-City, 567-0801 Osaka Japan; 9 Department of Rehabilitation Medicine, Wakayama Medical University, 811-1 Kimiidera,Wakayama City, 641-8509 Wakayama, Japan; 10 Department of Pharmacology, Arizona Health Sciences Center, University of Arizona, 1501 N. Campbell Avenue, 85724 Tucson, AZ USA

## Abstract

Emerging evidence suggests that the mesolimbic dopaminergic network plays a role in the modulation of pain. As chronic pain conditions are associated with hypodopaminergic tone in the nucleus accumbens (NAc), we evaluated the effects of increasing signaling at dopamine D1/D2-expressing neurons in the NAc neurons in a model of neuropathic pain induced by partial ligation of sciatic nerve. Bilateral microinjection of either the selective D1-receptor (Gs-coupled) agonist Chloro-APB or the selective D2-receptor (Gi-coupled) agonist quinpirole into the NAc partially reversed nerve injury-induced thermal allodynia. Either optical stimulation of D1-receptor-expressing neurons or optical suppression of D2-receptor-expressing neurons in both the inner and outer substructures of the NAc also transiently, but significantly, restored nerve injury-induced allodynia. Under neuropathic pain-like condition, specific facilitation of terminals of D1-receptor-expressing NAc neurons projecting to the VTA revealed a feedforward-like antinociceptive circuit. Additionally, functional suppression of cholinergic interneurons that negatively and positively control the activity of D1- and D2-receptor-expressing neurons, respectively, also transiently elicited anti-allodynic effects in nerve injured animals. These findings suggest that comprehensive activation of D1-receptor-expressing neurons and integrated suppression of D2-receptor-expressing neurons in the NAc may lead to a significant relief of neuropathic pain.

## Introduction

Parkinson’s disease is widely thought to be caused by a hypodopaminergic environment due to the degeneration of central dopaminergic neurons, and is associated with chronic pain in approximately 30–50% of patients [1, 2]. While there is no consensus regarding the essential mechanisms of this pain in Parkinson’s disease, it is believed that this hypodopaminergic environment may be critical for increased pain sensitivity.

It has been generally accepted that pain and emotion are intertwined since the ascending pain-associated network involves multiple brain regions that are responsible for both emotion and pain perception. The mesolimbic dopaminergic network that originates in the ventral tegmental area (VTA) is a critical player in a broad range of motivational, rewarding and pain events [3–7]. Mesolimbic dopaminergic dysfunction and/or hypodopaminergic conditions have been shown to be associated with neuropathic pain as well as reduced motivation and depression [8–10]. In our previous studies, we revealed that the intrinsic neuronal excitability of VTA-dopaminergic neurons projecting to the nucleus accumbens (NAc) was decreased in both neuropathic pain- and bone cancer pain-model mice [11]. Furthermore, we demonstrated that specific activation of VTA-NAc dopaminergic neurons using optogenetic techniques dramatically recovered the lowered pain threshold under conditions of neuropathic pain and bone cancer pain [11]. Human imaging studies have shown that the release of dopamine and the activity of dopamine receptors are reduced in the NAc of patients experiencing pain [12, 13]. A recent study provided further clear evidence that chronic pain negatively modulates mesolimbic dopaminergic neurons via a spinal-parabrachial-mesencephalic circuit [14]. Furthermore, it has also been documented that nerve injury increases the excitability of indirect spiny neurons located in the shell of the NAc [15].

Dopamine can activate postsynaptic dopamine D1- and D2-receptors in NAc neurons that are largely (i.e., approximately 95%) GABAergic medium spiny neurons (MSNs) [16]. The two types of MSNs in the NAc have been identified as dopamine D1-receptor-expressing MSNs (D1-MSNs) and dopamine D2-receptor-expressing MSNs (D2-MSNs) [17–19]. Although dopaminergic agonists have generally been associated with analgesia in human [20, 21], and microinjection of the dopamine D2 receptor agonist quinpirole into the NAc has been shown to inhibit the persistent phase of formalin-induced nociception in rats [22], little is known about the individual roles of D1-MSNs and D2-MSNs expressed in the NAc in the control of chronic pain.

The NAc region has been divided into three major subregions: the NAc medial shell (NAcMed), the NAc lateral shell (NAcLat) and the NAc core (NAcCo). Each of these subregions may have unique as well as common functions in the modulation of pain. Both D1-MSNs and D2-MSNs in these subregions could be endogenously and constantly regulated by the release of dopamine through Gs-coupled D1- and Gi-coupled D2-receptor-related intracellular signaling systems, respectively. In the present study, we determined if the microinjection of dopamine D1- and D2-receptor agonists or antagonists into each subregion of the NAc could produce relief of neuropathic pain in mice. Furthermore, by using optogenetic techniques to directly and specifically manipulate target MSN activities, we attempted to investigate whether selective stimulation of D1-receptor-expressing neurons and selective suppression of D2-receptor-expressing neurons, in the three subregions of the NAc could produce antinociceptive effects in mice.

## Method

### Animals

C57BL/6J (Tokyo Laboratory Animals Science Co., Ltd., Tokyo, Japan), wild type mice (C57BL/6N background) were either bred or purchase from the CLEA Japan (Tokyo, Japan). D1-tdTomato (B6.Cg-*Tg(Drd1a-tdTomato)6Calak*/J) (Stock #006785, The Jackson Laboratory, Bar Harbor, ME, USA), ChAT-cre (B6N.129S6(B6)-*Chat*^*tm2(cre)Lowl*^/J) (Stock #018957, Jackson Laboratory) mice and D2-tdTomato mice were used in the present study. C57BL/6-*Drd1a*^*tm1(cre)Phsh*^ (D1-cre) and C57BL/6-*Drd2*^*tm1(cre)Phsh*^ (D2-cre) mice were created at Cyagen Biosciences Inc. (Santa Clara, CA, USA). D2-tdTomato mice were obtained by breeding D2-cre mice with LSL-tdTomato (B6.Cg-*Gt(ROSA)26Sor*^*tm14(CAG−tdTomato)Hze*^/J) mice (Stock #007914, Jackson Laboratory).

Most of mice were 3–4 months of age at the start of behavioral experiments. All mice were housed at up to six mice per cage and kept in a temperature- and humidity-controlled room (24 ± 1 °C, 55 ± 5%, relative humidity) under a 12-h light-dark cycle (light on at 8:00 a.m. to 8:00 p.m.). Food and water were available *ad libitum* and behavioral experiments were performed in the light phase. All experiments were conducted in accordance with the Guide for Care and Use of Laboratory Animals of Hoshi University School of Pharmacy and Pharmaceutical Sciences, which is accredited by the Ministry of Education, Culture, Sports and Technology of Japan.

### Generation of D1-cre and D2-cre knock-in mice

C57BL/6-*Drd1a*^*tm1(cre)Phsh*^ (D1-cre) and C57BL/6-*Drd2*^*tm1(cre)Phsh*^ (D2-cre) mice, which were based on a C57BL/6 genetic background, were created at Cyagen Biosciences Inc. Two exons of *Drd1a* gene (NCBI Reference Sequence: NM_001291801.1), which is located on mouse chromosome 13, have been identified, with the ATG start codon in exon 2 and TGA atop codon in exon 2 (Transcript: Drd1-201 ENSMUST 00000021932.5). Eight exons of *Drd2* gene (NCBI Reference Sequence: NM_010077.2), which is located on mouse chromosome 9, have been identified, with the ATG start codon in exon 2 and TGA stop codon in exon 8 (Transcript: Drd2-001 ENSMUST 00000075764.7). To generate the 2 A-Cre knock-in at the mouse *Drd1a* or *Drd2* locus in C57BL/6 mice, the mixture of Cas9 mRNA, sgRNA, and Drd1-2A-Cre or Drd2-2A-Cre targeting vector, which can replace the TGA stop codon with 2A-Cre cassette according to CRISPR-Cas9 technology, was injected into mouse fertilized egg, then the eggs were transferred to surrogate mothers to obtain founder knock-in mice on the B6 background.

### Drugs

Chloro-APB hydrobromide (Sigma-Aldrich, St. Louis, MO, USA), quinpirole hydrochloride (Sigma-Aldrich) and (+)-SCH23390 hydrochloride (FUJIFILM Wako Pure Chemcal Corp., Osaka, Japan) were dissolved in saline (Otsuka Pharmaceutical Factory Inc., Tokushima, Japan). (S)-(-)-Sulpiride (Sigma-Aldrich) was dissolved in 0.1 N HCl, neutralized with 0.1 N NaOH to pH7, and adjusted to a final volume with saline. All drugs were locally administered into the medial and lateral shell in the NAc. The concentrations were adjusted to 6 µg/µL and 0.5µL/side was microinjected bilaterally. The concentration of drugs was basically chosen with reference to previous reports of rodent behaviors [23, 24].

### Clear, Unobstructed Brain/Body Imaging Cocktails and Computational analysis (CUBIC)

D1-tdTomato and D2-tdTomato mice were anesthetized with isoflurane (3%, inhalation) and transcardially perfused with 20 mL of PBS containing 10 U/mL heparin and then with 4% paraformaldehyde (PFA; FUJIFILM Wako Pure Chemical Corporation, Osaka, Japan) in 0.1 M phosphate buffer (PB, pH 7.4). The excised brain was post-fixed with the same fixative for 18–24 h at 4 °C. After being washed with PBS for 6 h (2 h × 3), the brain was immersed in PBS-diluted CUBIC-L (1:1) (Tokyo Chemical Industry Co., Ltd, Tokyo, Japan) and shaken gently (60 rpm, RT) for at least 8 h. On the next day, the brain was transferred to undiluted CUBIC-L and shaken overnight (60 rpm, RT). For whole-brain delipidation, the brain was immersed in fresh CUBIC-L and shaken gently (60 rpm, 37 °C) for 4 days. The CUBIC-L was changed every 1–2 days. On days 7 and 8, the brain was immersed and shaken overnight using PBS and PBS-diluted CUBIC-R^+^ (1:1) (Tokyo Chemical Industry), respectively. Finally, the brain was transferred to undiluted CUBIC-R^+^ and shaken gently (60 rpm, RT). Image-acquisition was performed using an UltraMicroscope II (Zoom body configuration) (Miltenyi Biotec B.V. & Co. KG, Bergisch Gladbach, Germany). 3D imaging analysis was conducted using an Imaris (Oxford Instruments, Abingdon, UK).

### Fluorescence immunohistochemistry

Mice were transcardially perfusion-fixed with 4% PFA in 0.1 M PB (pH 7.4) under anesthesia with isoflurane (3%, inhalation). The brain tissues were dissected after being post-fixed with 4% PFA and cryoprotected in 20-30 (w/v) % sucrose (FUJIFILM Wako Pure Chemical Corp.). The brain sections were embedded in an O.C.T. compound (Sakura Fine Technical, Tokyo, Japan), and the frozen sections were cut on a cryostat (10–20 μm) (CM1860; Leica Microsystems, Heidelberg, Germany). The Brain slices were blocked in 3% normal horse serum (NHS: Vector laboratories, Inc., Burlingame, CA, USA)/0.2% Triton X-100 in 0.01 M PBS for 1 h at RT. The brain slices were then incubated with goat anti-ChAT (1:100, Millipore) antibody for 48 h at 4°C . Following washes, they were incubated in donkey anti-goat Alexa 488 conjugated secondary antibody (1:400; Thermo Fischer Scientific) for 2 h at RT. The slices were mounted with Dako Fluorescence Mounting Medium (Dako, Glostrup, Denmark). Immunofluorescence was detected by a light microscope (BX-53; Olympus, Tokyo, Japan), and captured using a high-sensitivity digital CCD camera (MD-695; Molecular Devices, San Jose, CA, USA). Imaging analysis was performed using Metamorph 7.8 software (Molecular Devices).

### Virus

We synthesized AAV5-Flex-ChR2 (H134R)-mCherry and AAV5-Flex-ArchT-GFP. The final viral concentrations of AAV5-Flex-ChR2-mCherry and AAV5-Flex-ArchT-GFP were 2 × 10^13^ copies/mL. These aliquots of virus were stored at -80 °C until use.

### Virus injection and cannula implantation

Mice were placed in a stereotaxic apparatus (RWD Life Science, San Diego, CA, USA) under isoflurane (3%, inhalation) anesthesia, and the skull was exposed. A small hole was then made in the skull using a dental drill. Virus was bilaterally injected into the NAc core (from the bregma: AP + 1.4 mm, ML ± 1.5 mm, DV -3.6 mm from the brain surface at an angle of 10°: 1.0 µL was applied to each side using a Hamilton syringe or from the bregma: AP +1.4mm, ML ± 0.9 mm, DV -4.0 mm from the skull at an angle of 0°: 300 nL was applied to each side using a Nanoject III), the lateral shell (from the bregma: AP +1.0 mm, ML ± 1.8 mm, DV -4.9 mm from the skull at an angle of 0°: 300 nL was applied to each side using a Nanoject III) and the medial shell (from the bregma: AP +1.5 mm, ML ± 0.5 mm, DV -4.7 mm from the skull at an angle of 0°: 300 nL was applied to each side using a Nanoject III). More than 14 days after viral injection, a fiber cannula (EIM-330; Eicom, Kyoto, Japan) was implanted above the NAc core (from the bregma: AP + 1.4 mm, ML ± 1.5 mm, DV -3.1 mm from the brain surface at an angle of 10°), the lateral shell (from the bregma: AP +1.0 mm, ML ±1.8 mm, DV -4.4 mm from the skull at an angle of 0°), the medial shell (from the bregma: AP +1.5 mm, ML ± 1.4 mm, DV -4.2 mm from the skull at an angle of 10°) and VTA (from the bregma: AP -3.1 mm, ML ±1.3 mm, DV -4.2 mm from the skull at an angle of 10°) in mice expressing ChR2 or ArchT. The peek cannula (EIM-54; Eicom, Kyoto, Japan) was implanted into the lateral shell (from the bregma: AP +1.0mm, ML ± 1.8 mm, DV -4.9 mm from the skull at an angle of 0°) and the medial shell (from the bregma: AP +1.5 mm, ML ± 1.4 mm, DV -4.7 mm from the skull at an angle of 10°).

### Optical stimulation

Optical fibers (250 μm diameter; Lucir, Ibaraki, Japan) were placed in the fiber cannula. These fibers were connected to a 473 nm blue laser (COME2-LB473/100 model; Lucir) or 589 nm yellow laser (COME2-LY589/100 model; Lucir), and light pulses were generated through an electronic stimulator (Nihon Kohden, Tokyo, Japan). Mice expressing ChR2 were illuminated by blue light (473 nm, 30 Hz, 5 msec, 8 pulses every 5 s) for 30 min to control neuronal activity. Mice expressing ArchT were illuminated by continuous yellow light (589nm).

### Electrophysiological validation of ChR2 and ArchT activation

Coronal brain slices (250 μm) containing the NAc were prepared with a vibratome (VT-1200 S, Leica Biosystems, Wetzlar, Germany), using ice-cold cutting solution containing (in mM) 222.1 sucrose, 2.5 KCl, 1 CaCl_2_, 7 MgSO_4_, 1.4 NaH_2_PO_4_, 27 NaHCO_3_, and 0.5 ascorbic acid (oxygenated with 95% O_2_/5% CO_2_). Slices were recovered for at least 1 h at room temperature in oxygenated artificial cerebrospinal fluid containing (in mM) 128 NaCl, 3 KCl, 2 CaCl_2_, 2 MgCl_2_, 1.25 NaH_2_PO_4_, 24 NaHCO_3_, and 10 glucose. The fluorescence of the cells in the NAc was detected by an upright fluorescence microscope (ECLIPSE FN1; Nikon, Tokyo, Japan) using a 40× water-immersion objective and a sCMOS camera (Zyla 5.5 sCMOS; Andor Technology, Belfast UK). Whole-cell patch-clamp recording was made from D1- and D2-receptor-positive neurons expressing ChR2-mCherry and ArchT-GFP, respectively, with a Multiclamp 700B Amplifier (Molecular Devices, Sunnyvale, CA, USA). The recording electrodes were borosilicate glass pipettes (4–6 MΩ) filled with the following solution: (in mM): 120 potassium gluconate, 10 KCl, 10 HEPES, 10 phosphocreatine-Na_2_, 4 Mg_2_ATP, 0.3 Na_3_GTP, and 0.2 EGTA (pH 7.3 with KOH). Biocytin (0.2%) was included in the internal solution. Data were stored with pCLAMP 10 software (Molecular Devices). ChR2 and ArchT were excited with 450–495 nm and 540–600 nm light (XLED1; Excelitas Technologies, Waltham, MA, USA), respectively, delivered through the optical port of the microscope. After electrophysiological recording, slices were fixed with 4% paraformaldehyde at 4 °C and 24 h later incubated with streptoavidin-conjugated Alexa Fluor-350 (1:2,000; S11249, Thermo Fisher Scientific, Waltham, MA, USA) to identify the recorded cells.

### Neuropathic pain model

The neuropathic pain experiments were performed based on a previous report [25]. Before the experiments, we produced a partial sciatic nerve injury by tying a tight ligature with an 8-0 silk suture around approximately one-third to one-half the diameter of the sciatic nerve on the right side (ipsilateral side) in isoflurane (3%, inhalation)-anesthetized mice under a light microscope (SD30, Olympus, Tokyo, Japan).

### Thermal Paw-Withdrawal (Hargreaves) test and schedule for drug treatment

A thermal stimulus was created using a thermal stimulus apparatus (Model 7360; UGO BASILE, Varese, Italy) and applied to the plantar surface of the mouse’s hind paw to assess the thermal paw-withdrawal threshold as described previously [11]. Each measurement of drug-induced antinociception was performed on day 10 (Chloro-APB), day 11 (quinpirole), day 13 (SCH23390) and day 14 (sulpiride) after the ligation. We confirmed that pain threshold returned to the pre-treatment level just before each measurement for drugs.

### Statistical analysis

The data are presented as the mean ± SEM. All statistical analyses were performed with GraphPad Prism 8.0 (GraphPad Software, San Diego, CA, USA). The statistical significance of differences between the groups was assessed by a two-way analysis of variance followed by the Bonferroni multiple comparisons test. A p value of <0.05 was considered to reflect significance.

## Result

### Brain mapping of dopamine D1 or D2 receptor-expressing neurons in the NAc

**Fig. 1:**
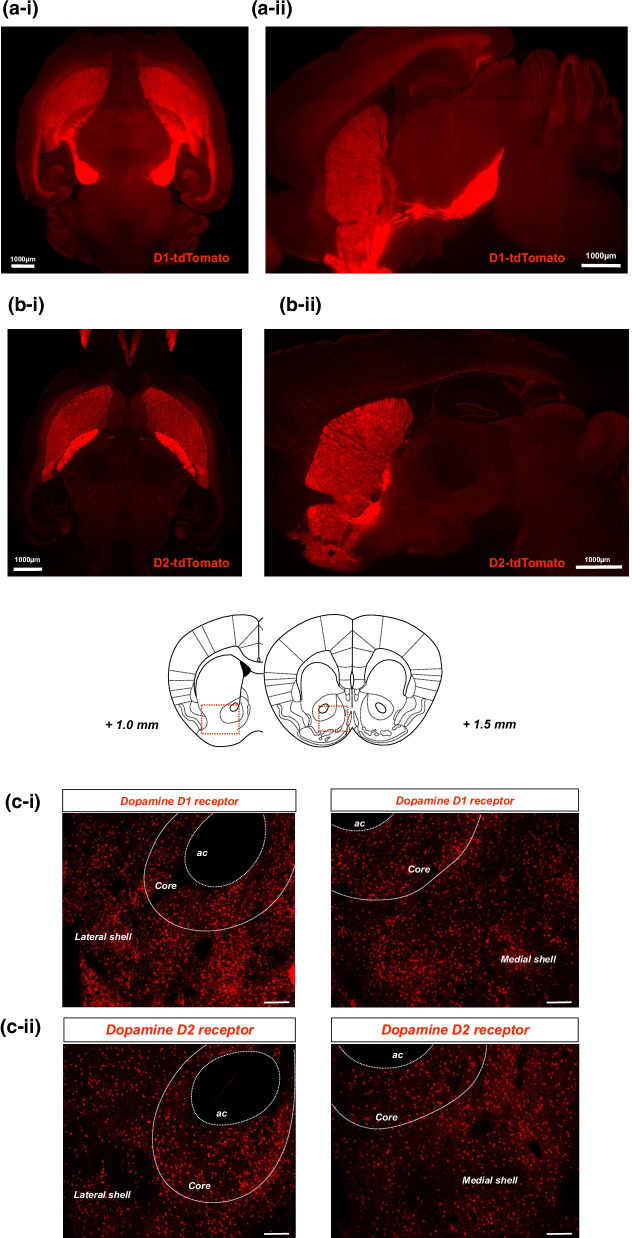
Brain mapping of dopamine D1 and D2 receptor-positive neurons. **a**, **b** Whole-brain imaging of D1 and D2 receptor-positive neurons of D1-tdTomato and D2-tdTomato mice, respectively. Fluorescent histochemical observation of D1 receptor-expressing neurons (**a**) or D2 receptor-expressing neurons (**b**) using CUBIC. Images were acquired from horizontal (**a**-i, **b**-i) and sagittal (**a**-ii, **b**-ii) sections. Scale bars = 1000 μm. **c** The distribution of D1 receptor-expressing (**c**-**i**) and D2 receptor-expressing (**c**-ii) neurons in the NAcLat and NacMed of D1-tdTomato or D2-tdTomato mice, respectively. The slices were not immunostained and mounted directly with Dako fluorescent mounting medium. Scale bars = 100 μm

Brain imaging of D1-tdTomato (Fig. 1a) and D2-tdTomato (Fig. 1b) mice using the “Clear, Unobstructed Brain/Body Imaging Cocktails and Computational analysis” (CUBIC) technique showed that both dopamine D1 receptor- and D2 receptor-positive cells were highly located in the NAc and striatum (Fig. 1a-i, b-i). Notably, the dopamine D1 receptor-positive cells in the NAc appeared to project into the midbrain region (Fig. 1a-ii), whereas no such projection could be identified in dopamine D2 receptor-positive neurons (Fig. 1b-ii). In the coronal section containing the NAc of D1-tdTomato mice, dopamine D1 receptor-expressing neurons were expressed not only in the core and lateral shell but also in the medial shell of the NAc (Fig. 1c-i). Dopamine D2 receptor-expressing neurons were also confirmed to be homogenously distributed in all three of the NAc subregions (Fig. 1c-ii).

### Effects of the administration of dopamine receptor agonists/antagonists into the NAc on neuropathic pain

**Fig. 2:**
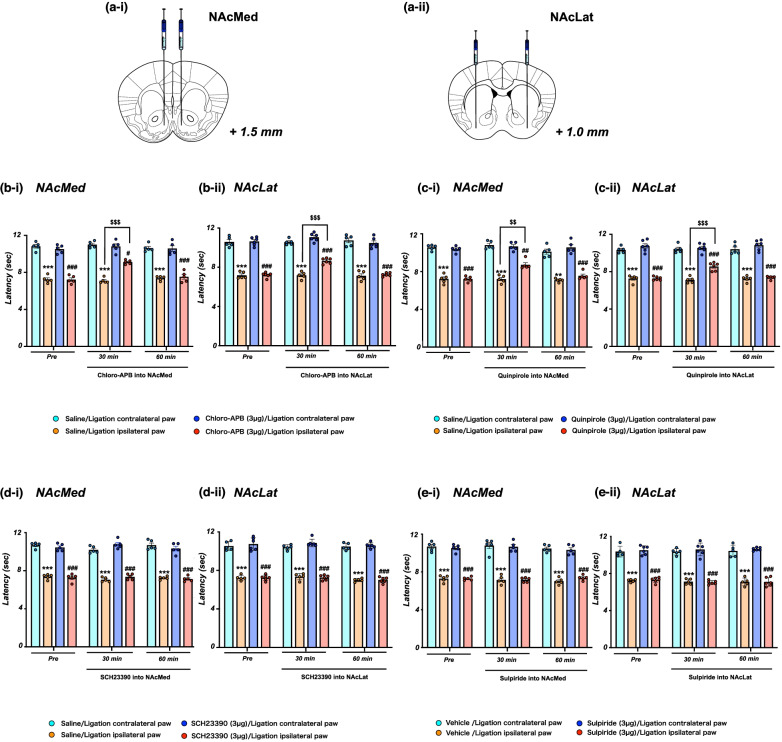
Effect of the injection of dopamine receptor agonists/antagonists into the NAc on neuropathic pain. **a** Schematic diagram showing the injection site of the NAcMed (**a**-i) or NAcLat (**a**-ii). **b** Latency in the response to thermal stimulation under a neuropathic pain-like state by administration of the selective dopamine D1 receptor agonist Chloro-APB into the NAcMed (**b**-i) and NAcLat (**b**-ii). Data are presented as the mean ± SEM of 5-6 animals. ^***^p < 0.001 vs. saline ligation contralateral paw; ^#^p < 0.05, ^###^p < 0.001 vs. Chloro-APB ligation contralateral paw; ^$$$^p < 0.001 vs. saline ligation ipsilateral paw. **c** Latency in the response to thermal stimulation under a neuropathic pain-like state by the administration of the selective dopamine D2 receptor agonist quinpirole into the NAcMed (**c**-i) and NAcLat (**c**-ii). Data are presented as the mean ± SEM of 5-6 animals. ^**^p < 0.01, ^***^p < 0.001 vs. saline ligation contralateral paw; ^##^p < 0.01, ^###^p < 0.001 vs. quinpirole ligation contralateral paw; ^$$^p < 0.01, ^$$$^< 0.001 vs. saline ligation ipsilateral paw. **d** Effects of the selective dopamine D1 receptor antagonist SCH23390 in the NAcMed and NAcLat in mice. Latency in the response to thermal stimulation under a neuropathic pain-like state by SCH23390 of the NAcMed (**d**-i) and NAcLat (**d**-ii). Data are presented as the mean ± SEM of 5-6 animals. ^***^p < 0.001 vs. saline ligation contralateral paw; ^###^p < 0.001 vs. SCH23390 ligation contralateral paw. **e** Latency in the response to thermal stimulation under a neuropathic pain-like state by the administration of dopamine D2 receptor antagonist sulpiride of the NAcMed (**e**-i) and NAcLat (**e**-ii). Data are presented as the mean ± SEM of 5-6 animals. ^***^p < 0.001 vs. vehicle ligation contralateral paw; ^###^p < 0.001 vs. sulpiride ligation contralateral paw

First, we examined the effect of microinjection of the selective dopamine D1 receptor agonist Chloro-APB (Fig. 2b-i, ^***^p < 0.001 vs. Saline/Ligation contralateral paw, ^#^p < 0.05, ^###^p < 0.001 vs. Chloro-APB (3 µg)/Ligation contralateral paw, ^$$$^p < 0.001 vs. Saline/Ligation ipsilateral paw) and the selective D2 receptor agonist quinpirole (Fig. 2c-i, ^**^p < 0.01, ^***^p < 0.001 vs. Saline/Ligation contralateral paw, ^##^p < 0.01, ^###^p < 0.001 vs. quinpirole (3 µg)/Ligation contralateral paw, ^$$^p < 0.01 vs. Saline/Ligation ipsilateral paw) into the NAcMed significantly restored the pain threshold that had been lowered under the neuropathic pain-like state. Furthermore, the decrease in the pain threshold due to sciatic nerve ligation was significantly and temporarily restored by the microinjection of Chloro-APB (Fig. 2b-ii, ^***^p < 0.001 vs. Saline/Ligation contralateral paw, ^###^p < 0.001 vs. Chloro-APB (3 µg)/Ligation contralateral paw, ^$$$^p < 0.001 vs. Saline/Ligation ipsilateral paw) and quinpirole (Fig. 2c-ii, ^***^p < 0.001 vs. Saline/Ligation contralateral paw, ^###^p < 0.001 vs. quinpirole (3 µg)/Ligation contralateral paw, ^$$$^p < 0.001 vs. Saline/Ligation ipsilateral paw) in the NAcLat. On the other hand, there was no recovery of the lowered pain threshold in mice with sciatic nerve ligation following microinjection of the selective dopamine D1 receptor antagonist SCH23390 or the selective dopamine D2 receptor antagonist sulpiride into either the NAcMed or NAcLat (Fig. 2d-i and d-ii, ^***^p < 0.001 vs. Saline/Ligation contralateral paw, ^###^p < 0.001 vs. SCH23390 (3 µg)/Ligation contralateral paw, Fig. 2e-i and e-ii, ^***^p < 0.001 vs. Vehicle/Ligation contralateral paw, ^###^p < 0.001 vs. Sulpiride (3 µg)/Ligation contralateral paw).

### Effects of the activation of D1-receptor-expressing neurons in the NAc on the lowered pain threshold due to neuropathic pain

**Fig. 3:**
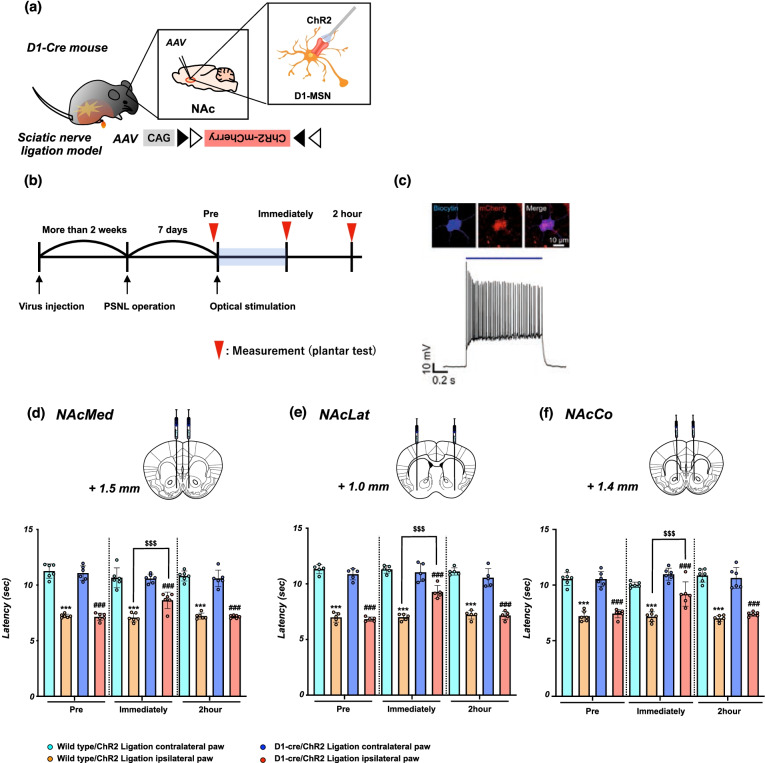
Effect of optical stimulation of D1-receptor-expressing neurons in the NAc on neuropathic pain. **a** Schematic showing that AAV-Flex-ChR2-mCherry was microinjected into the NAc of D1-cre mice. **b** Experimental timeline. **c** Biocytin-stained ChR2-mCherry-expressing neurons in a D1-cre mouse (upper) and representative current-clamp trace from a nucleus accumbens neuron expressing ChR2 showing action potentials in response to photoactivation (blue line) (lower). **d**-**f** Effects of optical stimulation of D1-receptor-expressing neurons in the NAcMed, NAcLat and NAcCo in mice. Latency in the response to thermal stimulation under a neuropathic pain-like state by optical stimulation of the NAcMed (**d**), NAcLat (**e**) and NAcCo (**f**) in Wild-type/ChR2 and D1-cre/ChR2 mice. Data are presented as the mean ± SEM of 5-6 animals. ^***^p < 0.001 vs. Wild-type/ChR2 ligation contralateral paw; ^###^p < 0.001 vs. D1-cre/ChR2 ligation contralateral paw; ^$$$^p < 0.001 vs. Wild-type/ChR2 ligation ipsilateral paw

To more precisely verify the role of D1-receptor-expressing neurons in the NAc in pain control, we generated transgenic mice (D1-cre/ChR2 mice) by the bilateral microinjection of AAV vector with a FLEX switch system to express ChR2 into the NAc of D1-Cre mice (Fig. 3a). Using these transgenic mice, we investigated whether the optical activation of D1-receptor-expressing neurons could change the pain threshold under a neuropathic pain-like state (Fig. 3b). We confirmed using electrophysiology that D1-ChR2-positive neurons in the NAc reliably responded to pulses of 470 nm blue light (Fig. 3c).

To investigate whether optical stimulation of D1-receptor-expressing neurons in the NAc could affect the changes in the pain threshold, partial nerve ligation was performed in D1-cre/ChR2 mice. Optical stimulation of D1-receptor-expressing neurons in either the NAcMed (Fig. 3d, ^***^p < 0.001 vs. Wild-type/ChR2 ligation contralateral paw; ^###^p < 0.001 vs. D1-cre/ChR2 ligation contralateral paw; ^$$$^p < 0.001 vs. Wild-type/ChR2 ligation ipsilateral paw) or NAcLat (Fig. 3e, ^***^p < 0.001 vs. Wild-type/ChR2 ligation contralateral paw; ^###^p < 0.001 vs. D1-cre/ChR2 ligation contralateral paw; ^$$$^p < 0.001 vs. Wild-type/ChR2 ligation ipsilateral paw) of D1-cre/ChR2 mice transiently, but significantly, reversed the lowered pain threshold due to neuropathic pain. As in the shell of the NAc, the lowering of the pain threshold by sciatic nerve ligation was temporarily but significantly restored by the optogenetic stimulation of D1-receptor-expressing neurons in the NAcCo of D1-cre/ChR2 mice (Fig. 3f, ^***^p < 0.001 vs. Wild-type/ChR2 ligation contralateral paw; ^###^p < 0.001 vs. D1-cre/ChR2 ligation contralateral paw; ^$$$^p < 0.001 vs. Wild-type/ChR2 ligation ipsilateral paw).

### Effects of the suppression of D2-receptor-expressing neurons in the NAc on the lowered pain threshold due to neuropathic pain

**Fig. 4:**
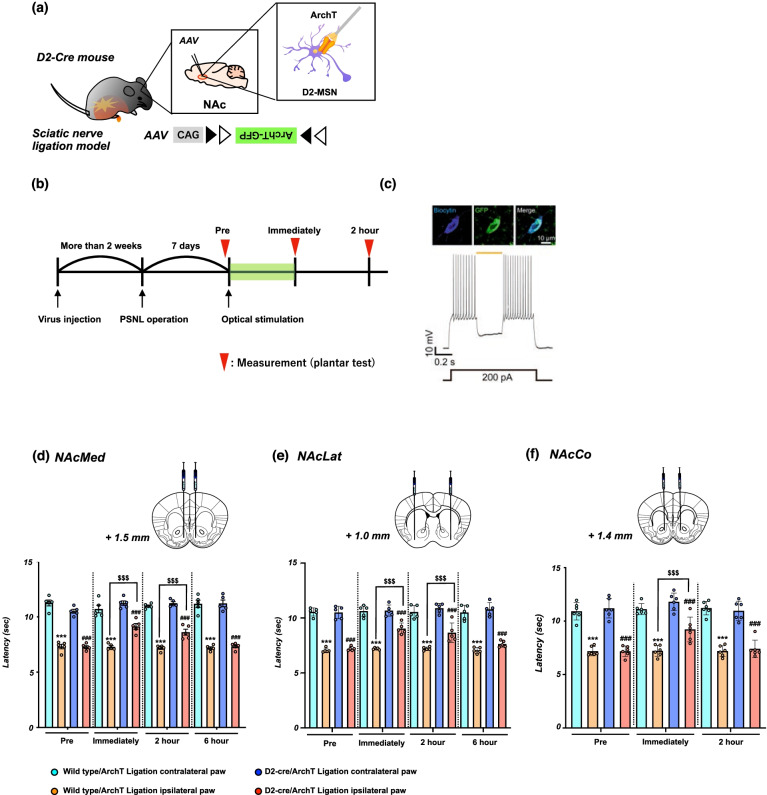
Effect of optical suppression of D2-receptor-expressing neurons in the NAc on neuropathic pain. **a** Schematic showing that AAV-Flex-ArchT-GFP was microinjected into the NAc of D2-cre mice. **b** Experimental timeline. **c** Examples of biocytin-stained ArchT-GFP-expressing neurons in a D2R-cre mouse (upper) and representative current-clamp trace from a NAc neuron expressing ArchT showing reliable suppression of the firing of neurons during photostimulation (orange line) (lower). **d**-**f** Effects of optical suppression of D2-receptor-expressing neurons in the NAcMed, NAcLat and NAcCo in mice. Latency in the response to thermal stimulation under a neuropathic pain-like state by optical stimulation of the NAcMed (**d**), NAcLat (**e**) and NAcCo (**f**) in Wild-type/ArchT and D2-cre/ArchT mice. Data are presented as the mean ± SEM of 5-6 animals. ^***^p < 0.001 vs. Wild-type/ArchT ligation contralateral paw; ^###^p < 0.001 vs. D2-cre/ArchT ligation contralateral paw; ^$$$^p < 0.001 vs. Wild-type/ArchT ligation ipsilateral paw

To more precisely identify the role of D2-receptor-expressing neurons in the NAc in pain control, we generated transgenic mice (D2-cre/ArchT mice) by microinjecting AAV vector with a FLEX switch system to express ArchT into the NAc of D2-Cre mice (Fig. 4a). Using these transgenic mice, we investigated whether the optical suppression of D2-receptor-expressing neurons could change the pain threshold under a neuropathic pain state (Fig. 4b). Electrophysiology experiments confirmed that D2-ArchT-positive neurons in the NAc reliably responded to 589 nm yellow light (Fig. 4c). Partial nerve ligation was performed to investigate changes in the pain threshold by optical suppression of D2-receptor-expressing neurons in the NAc of these transgenic mice. As a result, specific suppression of D2-receptor-expressing neurons in either the NAcMed (Fig. 4d, ^***^p < 0.001 vs. Wild-type/ArchT ligation contralateral paw; ^###^p < 0.001 vs. D2-cre/ArchT ligation contralateral paw; ^$$$^p < 0.001 vs. Wild-type/ArchT ligation ipsilateral paw) or NAcLat (Fig. 4e, ^***^p < 0.001 vs. Wild-type/ArchT ligation contralateral paw; ^###^p < 0.001 vs. D2-cre/ArchT ligation contralateral paw; ^$$$^p < 0.001 vs. Wild-type/ArchT ligation ipsilateral paw) in D2-cre/ArchT mice significantly restored the lowered threshold due to neuropathic pain. Furthermore, the lowered threshold following sciatic nerve ligation was transiently, but significantly, reversed by the optogenetic suppression of D2-receptor-expressing neurons in the NAcCo of D2-cre/ArchT mice (Fig. 4f, ^***^p < 0.001 vs. Wild-type/ArchT ligation contralateral paw; ^###^p < 0.001 vs. D2-cre/ArchT ligation contralateral paw; ^$$$^p < 0.001 vs. Wild-type/ArchT ligation ipsilateral paw).

### Effect of optical stimulation of D1-receptor-expressing neurons in the NAc projecting to the VTA on neuropathic pain

**Fig. 5:**
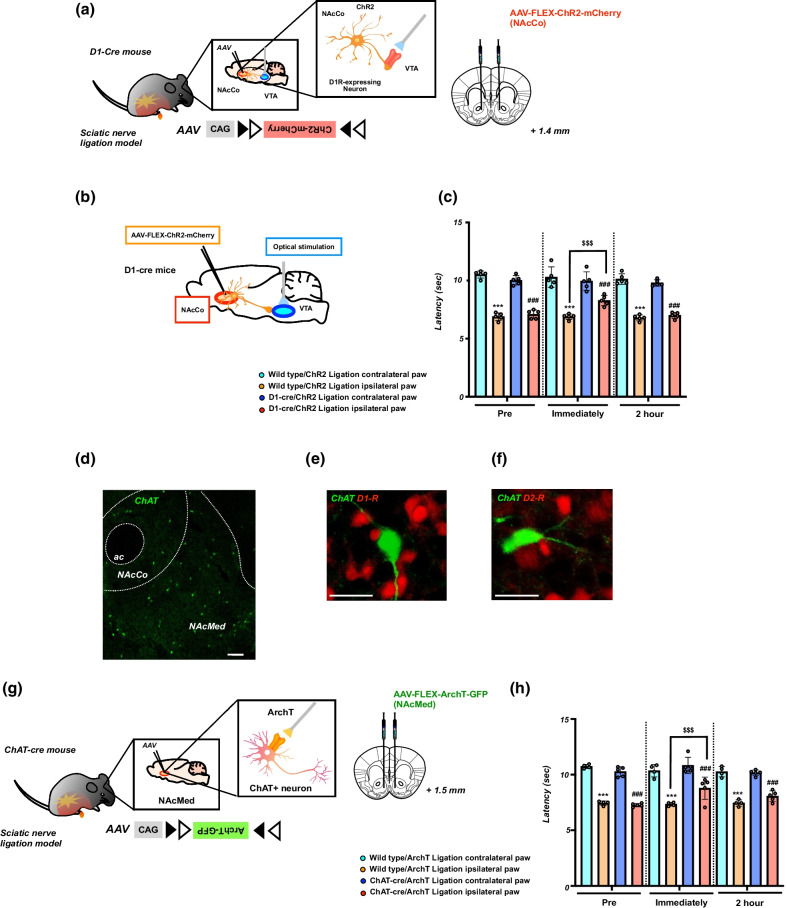
Effect of optical stimulation of D1-receptor-expressing neurons in the NAc projecting to the VTA and optical inhibition of cholinergic neurons in the NAc on neuropathic pain. **a** Schematic showing that AAV-Flex-ChR2-mCherry was microinjected into the NAcCo of D1-cre mice. **b** Schematic diagram showing the experimental design. **c** Effects of optical stimulation of D1-receptor-expressing neurons projecting to the VTA. Latency in the response to thermal stimulation under a neuropathic pain-like state by optical stimulation of the VTA in Wild-type/ChR2 and D1-cre/ChR2 mice. Data are presented as the mean ± SEM of 5 animals. ^***^p < 0.001 vs. Wild-type/ChR2 ligation contralateral paw; ^###^p < 0.001 vs. D1-cre/ChR2 ligation contralateral paw; ^$$$^p < 0.001 vs. Wild-type/ChR2 ligation ipsilateral paw. **d** Immunohistochemical staining images showing choline acetyltransferase (ChAT)-positive neuron in the NAc. Scale bar = 200 μm. **e**, **f** The images showing ChAT (green) and dopamine D1 receptor (red) of D1-tdTomato mice (**e**) or dopamine D2 receptor (red) of D2-tdTomato mice (**f**). Scale bar = 25 μm. **g** Schematic showing that AAV-Flex-ArchT-GFP was microinjected into the NAcMed of D1-cre mice. **h** Effects of the optical suppression of ChAT^+^ neurons in the NAcMed. Latency in the response to thermal stimulation under a neuropathic pain-like state by optical suppression of ChAT^+^ neurons in Wild-type/ArchT and ChAT-cre/ArchT mice. Data are presented as the mean ± SEM of 4–5 animals. ^***^p < 0.001 vs. Wild-type/ArchT ligation contralateral paw; ^###^p < 0.001 vs. ChAT-cre/ArchT ligation contralateral paw; ^$$$^p < 0.001 vs. Wild-type/ArchT ligation ipsilateral paw

It has been documented that some D1-MSNs located within the NAc terminals mainly synapse onto VTA GABA neurons [26]. Therefore, we infected the NAcCo of D1-cre mice with the AAV vector expressing ChR2 (Fig. 5a, b) and investigated changes in the pain threshold by optical activation of terminals of D1-receptor-expressing neurons projecting to the VTA. As a result, optical activation of terminals of D1-receptor-expressing neurons in the VTA in D1-cre/ChR2 mice also partly, but significantly, recovered the lowered pain threshold in mice with sciatic nerve ligation (Fig. 5c, ^***^p < 0.001 vs. Wild-type/ChR2 ligation contralateral paw; ^###^p < 0.001 vs. D1-cre/ChR2 ligation contralateral paw; ^$$$^p < 0.001 vs. Wild-type/ChR2 ligation ipsilateral paw).

### Effect of optical suppression of cholinergic interneurons in the NAc on neuropathic pain

It has been demonstrated that choline acetyltransferase (ChAT)-positive (i.e., cholinergic) interneurons negatively modulate D1-MSNs in the NAc, whereas they positively regulate D2-MSNs in this area [27, 28]. To confirm the presence of ChAT-positive cholinergic interneurons in the NAc, we performed immunohistochemistry. As a result, ChAT immunoreactivity was detected mostly in the NAcMed (Fig. 5d). In the NAcMed of D1-tdTomato or D2-tdTomato mice, ChAT-positive neurons were distributed adjacent to either D1- or D2-receptor-expressing neurons (Fig. 5e, f). Therefore, we infected the NAcMed of ChAT-cre mice with the AAV vector expressing ArchT (Fig. 5g) and investigated changes in the pain threshold by optical suppression of cholinergic interneurons in the NAcMed. As a result, the optical suppression of cholinergic interneurons in the NAcMed of ChAT-cre/ArchT mice significantly reversed the lowered thresholds in mice with sciatic nerve ligation (Fig. 5h, ^***^p < 0.001 vs. Wild-type/ArchT ligation contralateral paw; ^###^p < 0.001 vs. ChAT-cre/ArchT ligation contralateral paw; ^$$$^p < 0.001 vs. Wild-type/ArchT ligation ipsilateral paw).

## Discussion

A growing body of evidence suggests that the NAc, which is one of the major terminals of VTA dopaminergic neurons, is critically involved in not only emotional functions and reward-related behaviors, but also pain control [3, 29, 30]. Since most neurons in the NAc express either D1 or D2 receptors [17–19], we investigated the functional role of D1-receptor-expressing neurons and D2-receptor-expressing neurons located in the NAc in relief of experimental neuropathic pain. We first confirmed that bilateral microinjection of either the selective D1-receptor agonist Chloro-APB or the selective D2-receptor agonist quinpirole into the NAcMed and NAcLat, both of which highly expressed D1- and D2-receptors, transiently, but significantly, reversed the reduction in the pain threshold in mice with partial sciatic nerve ligation. On the other hand, bilateral microinjection of the selective D1-receptor antagonist SCH23390 or the selective D2-receptor antagonist sulpiride into both the NAcMed and NAcLat had no effect on this reduced pain threshold. Similar to these results, we then demonstrated for the first time that optical stimulation of D1-receptor-expressing neurons in either the NAcMed, NAcLat or NAcCo, transiently, but significantly, suppressed the neuropathic pain-like state in mice, whereas optical suppression of D2-receptor-expressing neurons in these regions of the NAc restored the reduced pain threshold under a state of neuropathic pain. These findings are also consistent with outcomes of dopamine receptor modulation in other models of hyperalgesia. Unilateral microinjection of SCH23390 into the NAc suppressed the antinociceptive effects induced by morphine in morphine-sensitized rats [31]. Furthermore, antinociception induced by intra-basolateral amygdala administration of WIN55,212-2, a cannabinoid receptor agonist, has been shown to be prevented by microinjection of SCH23390 into the NAc [32]. It has also been documented that intra-VTA injection of orexin A-induced antinociception is suppressed by intra-NAc infusions of SCH23390 [33]. Taken together, the present findings provide compelling evidence that either activation of D1-receptor-expressing neurons (probably D1-MSNs) or suppression of D2-receptor-expressing neurons (probably D2-MSNs) in the NAcMed, NAcLat and NAcCo can elicit pain relief.

It has been reported that a significant number of D1-MSNs, but not D2-MSNs, in the NAc project directly to midbrain regions, the VTA and substantia nigra [34, 35]. In the VTA, most of the nerve terminals of D1-MSNs from the NAc have been shown to be directly connected to GABAergic interneurons [26], and activation of these projecting D1-MSNs leads to indirect activation of VTA-dopaminergic neurons through the disinhibition of GABAergic neurons [26]. In the present study, we found that the optical stimulation of D1-receptor-expressing neurons in the NAc projecting to the VTA partly, but significantly, recovered the decrease in the pain threshold in mice with partial sciatic nerve ligation. These findings raise intriguing possibilities that the NAc-VTA feedforward dopaminergic circuits via D1-MSNs could contribute to anti-pain and anti-negative emotion systems for maintaining homeostasis in the setting of chronic pain.

Even more so than we had expected, we found that optical suppression of cholinergic interneurons in the NAc, which comprise approximately 1-2% of the total neurons in this area [16], slightly, but significantly, recovered the lowering of the pain threshold under a state of neuropathic pain. As noted above, the results of the present study showed that specific inhibition of D2-receptor-expressing neurons in the NAc partly restored the lowered pain thresholds due to neuropathic pain. Current evidence has suggested that cholinergic interneurons in the NAc that have GABAergic input from the VTA are directly connected to D1-MSNs [27] and D2-MSNs [28, 36]. Under physiological conditions, the release of acetylcholine from these cholinergic interneurons has been shown to negatively and positively modulate the activity of D1-MSNs and D2-MSNs via muscarinic M4 and M1 receptors, respectively [28, 37]. In the present study, we confirmed that ChAT-positive neurons in the NAc region were located adjacent to either D1- or D2-receptor-expressing neurons. Taken together, the present behavioral findings indicate that the restoration of the decreased pain threshold due to neuropathic pain by the inhibition of cholinergic interneurons in the NAc may partly result from indirect inhibition of D2-MSNs as well as possible activation of D1-MSNs in the NAc. Furthermore, although cholinergic interneurons in the NAc have been also shown to express dopamine D2 receptors [38], the fact that dopamine D2 receptors in the NAc are mostly expressed in D2-MSNs suggests that the present finding may result from the effect of D2-MSNs. However, further research is still required on this point.

We need to consider the present fact that independent manipulation of D1- or D2-receptor-expressing neurons produced the weak antinociceptive effect against the thermal hyperalgesia. Although mechanical allodynia or ongoing pain instead of only one outcome measurement should be evaluated under the present condition, we propose that the present results may reflect from the fact that we didn’t manipulate both D1- and D2-receptor expressing neurons together.

In conclusion, we demonstrated here that the lowered pain thresholds due to partial sciatic nerve ligation was partly recovered by activation of D1-receptor-expressing neurons and suppression of D2-receptor-expressing neurons in the NAc using optogenetic techniques to specifically manipulate the target neurons. These findings suggest that activation of D1-MSNs and suppression of D2-MSNs in the NAc may be a unique and useful approach to relieve neuropathic pain.

## Data Availability

All of the data generated and analyzed in this study are included in this published article.

## Abbreviations

AAV: Adeno-associated virus
ArchT: Archaerhodopsin
ChAT: Choline acetyltransferase
ChR2: Channelrhodopsin-2
CUBIC: Clear, Unobstructed Brain/Body Imaging Cocktails and Computational analysis
D1-MSNs: Dopamine D1 receptor-expressing medium spiny neurons
D2-MSNs: Dopamine D2 receptor-expressing medium spiny neurons
GABA: Gamma-amino butyric acid
MSNs: Medium spiny neurons
NAc: Nucleus accumbens
NAcCo: NAc Core
NAcLat: NAc Lateral shell
NAcMed: NAc Medial shell
VTA: Ventral tegmental area

## References

[1] Conte A, Khan N, Defazio G, Rothwell JC, Berardelli A (2013). Pathophysiology of somatosensory abnormalities in Parkinson disease. Nat Rev Neurol.

[2] Fil A, Cano-de-la-Cuerda R, Muñoz-Hellín E, Vela L, Ramiro-González M, Fernández-de-Las-Peñas C (2013). Pain in Parkinson disease: a review of the literature. Parkinsonism Relat Disord.

[3] Navratilova E, Porreca F (2014). Reward and motivation in pain and pain relief. Nat Neurosci.

[4] Pignatelli M, Bonci A (2015). Role of Dopamine neurons in reward and aversion: a synaptic plasticity perspective. Neuron.

[5] Schultz W (2016). Doapmine reward prediction error coding. Dialogues Clin Neurosci.

[6] Pignatelli M, Umanah GKE, Ribeiro SP, Chen R, Karuppagounder SS, Yau HJ, Eacker S, Dawson VL, Dawson TM, Bonci A (2017). Synaptic plasticity onto dopamine neurons shapes fear learning. Neuron.

[7] Watabe-Uchida M, Eshel N, Uchida N (2017). Neural circuitry of reward prediction error. Annu Rev Neurosci.

[8] Nestler EJ, Carlezon WA (2006). The mesolimbic dopamine reward circuit in depression. Biol Psychiatry.

[9] Ren W, Centeno MV, Berger S, Wu Y, Na X, Liu X, Kondapalli J, Apkarian AV, Martina M, Surmeier DJ (2016). The indirect pathway of the nucleus accumbens shell amplifies neuropathic pain. Nat Neurosci.

[10] Dubol M, Trichard C, Leroy C, Sandu AL, Rahim M, Granger B, Tzavara ET, Karila L, Martinot JL, Artiges E (2018). Dopamine transporter and reward anticipation in a dimensional perspective: a multimodal brain imaging study. Neuropsychopharmacology.

[11] Watanabe M, Narita M, Hamada Y, Yamashita A, Tamura H, Ikegami D, Kondo T, Shinzato T, Shimizu T, Fukuchi Y, Muto A, Okano H, Yamanaka A, Tawfik VL, Kuzumaki N, Navratilova E, Porreca F, Narita M (2018). Activation of ventral tegmental area dopaminergic neurons reverses pathological allodynia resulting from nerve injury or bone cancer. Mol Pain.

[12] Baliki MN, Geha PY, Fields HL, Apkarian AV (2010). Predicting value of pain and analgesia:nucleus accumbens response to noxious stimuli changes in the presence of chronic pain. Neuron.

[13] Martikainen IK, Nuechterlein EB, Peciña M, Love TM, Cummiford CM, Green CR, Stohler CS, Zubieta JK (2015). Chronic back pain is associated with alterations in dopamine neurotransmission in the ventral striatum. J Neurosci.

[14] Yang H, de Jong JW, Cerniauskas I, Peck JR, Lim BK, Gong H, Fields HL, Lammel S (2021). Pain modulates dopamine neurons via a spinal-parabrachial-mesencephalic circuit. Nat Neurosci.

[15] Ren W, Centeno MV, Wei X, Wickersham I, Martina M, Apkarian AV, Surmeier DJ (2021). Adaptive alterations in the mesoaccumbal network after peripheral nerve injury. Pain.

[16] Robison AJ, Nestler EJ (2011). Transcriptional and epigenetic mechanisms of addiction. Nat Rev Neurosci.

[17] Gerfen CR, Engber TM, Mahan LC, Susel Z, Chase TN, Monsma FJ (1990). Sibley DR. D1 and D2 dopamine receptor-regulated gene expression of striatonigral and striatopallidal neurons. Science.

[18] Surmeier DJ, Song WJ, Yan Z (1996). Coordinated expression of dopamine receptors in neostriatal medium spiny neurons. J Neurosci.

[19] Kreitzer AC, Malenka RC (2008). Striatal plasticity and basal ganglia circuit function. Neuron.

[20] Hagelberg N, Jääskeläinen SK, Martikainen IK, Mansikka H, Forssell H, Scheinin H, Hietala J, Pertovaara A (2004). Striatal dopamine D2 receptors in modulation of pain in humans: a review. Eur J Pharmacol.

[21] Potvin S, Grignon S, Marchand S (2009). Human evidence of a supra-spinal modulating role of dopamine on pain perception. Synapse.

[22] Taylor BK, Joshi C, Uppal H (2003). Stimulation of dopamine D2 receptors in the nucleus accumbens inhibits inflammatory pain. Brain Res.

[23] Nazari-Serenjeh F, Zarrabian S, Azizbeigi R, Haghparast A (2021). Effects of dopamine D1- and D2-like receptors in the CA1 region of the hippocampus on expression and extinction of morphine-induced conditioned place preference in rats. Behav Brain Res.

[24] Ouyang J, Carcea I, Schiavo JK, Jones KT, Rabinowitsch A, Kolaric R, Cabeza de Vaca S, Froemke RC, Carr KD (2017). Food restriction induces synaptic incorporation of calcium-permeable AMPA receptors in nucleus accumbens. Eur J Neurosci.

[25] Seltzer Z, Dubner R, Shir Y (1990). A novel behavioral model of neuropathic pain disorders produced in rats by partial sciatic nerve injury. Pain.

[26] Bocklisch C, Pascoli V, Wong JC, House DR, Yvon C, de Roo M, Tan KR, Lüscher C (2013). Cocaine disinhibits dopamine neurons by potentiation of GABA transmission in the ventral tegmental area. Science.

[27] Mamaligas AA, Ford CP (2016). Spontaneous synaptic activation of muscarinic receptors by striatal cholinergic neuron firing. Neuron.

[28] Francis TC, Yano H, Demarest TG, Shen H, Bonci A (2019). High-Frequency activation of nucleus accumbens d1-MSNs drives excitatory potentiation on D2-MSNs. Neuron.

[29] Ambroggi F, Ghazizadeh A, Nicola SM, Fields HL (2011). Roles of nucleus accumbens core and shell in incentive-cue responding and behavioral inhibition. J Neurosci.

[30] Makary MM, Polosecki P, Cecchi GA, DeAraujo IE, Barron DS, Constable TR, Whang PG, Thomas DA, Mowafi H, Small DM, Geha P (2020). Loss of nucleus accumbens low-frequency fluctuations is a signature of chronic pain. Proc Natl Acad Sci U S A.

[31] Reisi Z, Bani-Ardalan M, Zarepour L, Haghparast A (2014). Involvement of D1/D2 dopamine receptors within the nucleus accumbens and ventral tegmental area in the development of sensitization to antinociceptive effect of morphine. Pharmacol Biochem Behav..

[32] Haghparast A, Ghalandari-Shamami M, Hassanpour-Ezatti M (2012). Blockade of D1/D2 dopamine receptors within the nucleus accumbens attenuated the antinociceptive effect of cannabinoid receptor agonist in the basolateral amygdala. Brain Res.

[33] Yazdi-Ravandi S, Razavi Y, Haghparast A, Goudarzvand M, Haghparast A (2014). Orexin A induced antinociception in the ventral tegmental area involves D1 and D2 receptors in the nucleus accumbens. Pharmacol Biochem Behav.

[34] Lu XY, Churchill L, Kalivas PW (1997). Expression of D1 receptor mRNA in projections from the forebrain to the ventral tegmental area. Synapse.

[35] Gerfen CR, Surmeier DJ (2011). Modulation of striatal projection systems by dopamine. Annu Rev Neurosci.

[36] Al-Hasani R, Gowrishankar R, Schmitz GP, Pedersen CE, Marcus DJ, Shirley SE, Hobbs TE, Elerding AJ, Renaud SJ, Jing M, Li Y, Alvarez VA, Lemos JC, Bruchas MR (2021). Ventral tegmental area GABAergic inhibition of cholinergic interneurons in the ventral nucleus accumbens shell promotes reward reinforcement. Nat Neurosci.

[37] Bernard V, Normand E, Bloch B (1992). Phenotypical characterization of the rat striatal neurons expressing muscarinic receptor genes. J Neurosci.

[38] Alcantara AA, Chen V, Herring BE, Mendenhall JM, Berlanga ML (2003). Localization of dopamine D2 receptors on cholinergic interneurons of the dorsal striatum and nucleus accumbens of the rat. Brain Res.

